# Genotype-dependent Burst of Transposable Element Expression in Crowns of Hexaploid Wheat (*Triticum aestivum L.*) during Cold Acclimation

**DOI:** 10.1155/2012/232530

**Published:** 2012-02-28

**Authors:** Debbie Laudencia-Chingcuanco, D. Brian Fowler

**Affiliations:** ^1^Genomics and Gene Discovery Unit, USDA-ARS WRRC, 800 Buchanan Street, Albany, CA 94710, USA; ^2^Department of Plant Sciences, University of Saskatchewan, 51 Campus Drive, Saskatoon, SK, Canada S7N 5A8

## Abstract

The expression of 1,613 transposable elements (TEs) represented in the Affymetrix Wheat Genome Chip was examined during cold treatment in crowns of four hexaploid wheat genotypes that vary in tolerance to cold and in flowering time. The TE expression profiles showed a constant level of expression throughout the experiment in three of the genotypes. In winter Norstar, the most cold-hardy of the four genotypes, a subset of the TEs showed a burst of expression after vernalization saturation was achieved. About 47% of the TEs were expressed, and both Class I (retrotransposons) and Class II (DNA transposons) types were well represented. *Gypsy* and *Copia* were the most represented among the retrotransposons while *CACTA* and *Mariner* were the most represented DNA transposons. The data suggests that the *Vrn-A1* region plays a role in the stage-specific induction of TE expression in this genotype.

## 1. Introduction

Transposable elements (TEs) are DNA sequences that can move or transpose to new locations within the genome. Transposable elements are generally classified based on their mechanism of transposition. Class I TE, or retrotransposons, move by a “copy and paste” mechanism whereby the TE is transcribed into an RNA intermediate that is converted to DNA by RNA-dependent DNA polymerases before it reinserts itself in the genome. Class II TE, or DNA transposons, utilize the “cut and paste” mechanism wherein the TE element DNA itself is excised from the genome and reinserted in a new position. TE transposition has been shown to generate mutations that can alter gene expression, and to create gene deletions and duplications, chromosome breaks, and genome rearrangements [1]. Thus, TEs can be a powerful force for species adaptation to adverse biotic and abiotic challenges and a facilitator of speciation by creating potentially advantageous genetic variations upon which natural selection can act [2, 3]. 

 Transposable elements are ubiquitous and can be found in both prokaryotic and eukaryotic genomes. In plants (especially cereals), TEs make up a large portion of the genome. In wheat, repetitive and transposable elements comprise more than 80% of the genomic sequence [4]. To prevent the potentially harmful effects of TE transposition, the host plant has evolved several mechanisms to repress TE expression [5, 6]. TE can be silenced before or after transcription. Transcriptional silencing of TE includes DNA methylation and chromatin remodeling, which renders the elements unavailable for transcription. On the other hand, posttranscription silencing involves small noncoding RNA (sRNA) which directs sequence-specific degradation of transcripts.

 Several events, which cause what Barbara McClintock called “genome shock,” have been shown to release TEs from repression [1, 7]. In plants, these activating events include genome hybridization [8], nutrient deprivation [9], infection [10, 11], and abiotic stresses like drought [12] and high temperature [10, 13]. One report showed a cold-induced activation of a family of retrotransposons in alfalfa [14]. In this paper we explored the response of TEs in wheat in response to cold.

We previously carried out a genome-wide expression analysis of the response to cold treatment of the four hexaploid wheat genotypes considered in this study [15]. In these genotypes, *Vrn-A1*, a major regulator of the transition of the shoot apex from vegetative to reproductive meristem in response to cold treatment in wheat, was swapped between a highly cold tolerant line, winter Norstar, and a cold-sensitive cultivar spring Manitou (see Table 1). Allelic variations in the *Vrn-A1* locus are the main determinants of the winter and spring habit in wheat [16, 17]. The swapped region is less than 37 cM and does not include *Frost resistance-2 *(*Fr-2*), a major locus that controls tolerance to low temperature located about 40 cM proximal to *Vrn-A1 *in chromosome 5AL [18]. We reported the identification of 2771 differentially expressed genes that could be involved in the development of low-temperature tolerance [15]. We have now mined this same dataset to investigate the expression of transposable elements during cold treatment in hexaploid wheat. We show that members of both Class I and Class II types of transposable elements in wheat are expressed during cold treatment. Furthermore, we showed that a genotype-dependent burst of TE expression in the crown occurs after vernalization saturation has been achieved and implicates the winter *vrn-A1* region to play a role in this event.

## 2. Materials and Methods

### 2.1. Plant Materials and Growth Conditions

Wheat (*Triticum aestivum *L.) cultivars and near isogenic lines (NILs) used in this study were developed and characterized as described in a previous microarray experiment [15, 18]. The lines differ in tolerance to low temperature and in flowering time. The major vernalization locus, *Vrn-A1*, which is a major determinant of flowering time in wheat, was swapped in the winter cultivar “Norstar” (*vrn-A1*) and the spring cultivar “Manitou” (*Vrn-A1*). The initial hybrid was backcrossed to each parent ten times prior to selfing to produce homozygous NILs (spring Norstar and winter Manitou) that are theoretically ~99.9% genetically similar to the parental lines. For each backcross, a phenotype-based selection of progeny with *Vrn-A1 *or* vrn-A1* locus was used to ensure that the donor parent allele was incorporated into the genetic background of the recurrent parent.

For these studies, seeds were imbibed in the dark for 2 days at 4°C and then transferred to an incubator and allowed to germinate for 3 days at 22°C. Actively germinating seeds were transferred, embryo down, to plexiglass trays with holes backed by a 1.6 mm mesh screen and grown for 10 days in hydroponic tanks filled with continuously aerated one-half strength modified Hoagland's solution [19] at 20°C in 16-hour days at 320 *μ*mol m^−2^ s^−1^ PPFD, by which time they had 3-4 fully developed leaves. The seedlings were then transferred to 6°C chambers (measured at crown level) under 16-hour photoperiod and 220 *μ*mol m^−2^ s^−1^ PPFD and sampled at regular intervals. Details on phenological development and cold acclimation of these lines when grown under these conditions have been given in an earlier publication [15].

### 2.2. Global Gene Expression Profiling

 The microarray study was performed as previously reported [15]. The experimental design included 4 genotypes (winter and spring Norstar NIL and spring and winter Manitou NIL) acclimated at 0, 2, 14, 21, 35, 42, 56, and 70 days. Crown tissue was harvested after each acclimation period at the same time each day (4 hours after dawn) to neutralize circadian rhythm effect. RNA was isolated from three biological replicate samples (pool of 25 crowns/sample) for each acclimation period. RNA labelling and Affymetrix Wheat Genome GeneChip array hybridization were performed according to the manufacturer's instructions (Affymetrix Inc, Santa Clara, CA, USA). Microarray data were extracted from scanned GeneChip images and analyzed using the Affymetrix Microarray Suite (MAS) version 5.0. Probe set signal normalization and summarization was carried out using the Robust Multi-array normalization algorithm (RMA), as implemented in GeneSpring software (Agilent, Santa Clara, CA, USA). RMA-normalized values were filtered for those present (as determined by the Affymetrix MAS probe summarization protocol) in 2 out of 3 of the biological replicates for each time point. In this paper, a probe set will be deemed to represent a potentially unique wheat gene or gene encoding a transposable element. The accumulation of its transcripts as measured by the signal intensities in each probe set represents the “expression” of the gene. Clustering of gene expression profiles using the *k*-means algorithm was implemented in the software Genesis [20]. The most recent annotations for the probesets were verified using wheat data in Plant Expression Database (http://www.plexdb.org/). Microarray data have been deposited to the NCBI Gene Expression Omnibus (GEO) database with accession number GSE23889.

### 2.3. Probesets for Wheat Transposable Elements

 The sequences of transposable elements identified from wheat were downloaded from the Triticeae Repeat Element (TREP; release #10, July 10, 2008) database in GrainGenes (http://wheat.pw.usda.gov/ITMI/Repeats/) and used to query the target sequences used to generate the Affymetrix Wheat Genome Chip using BLASTN [21].

### 2.4. TE Expression during Drought Stress

 The RMA-normalized data (TA23_RMA_tmt_mean.txt) for the genome-wide expression of wheat genes during drought stress using the Affymetrix Wheat Genome GeneChip [12] was downloaded from the Plant Expression Database (http://www.plexdb.org/). 

## 3. Results

### 3.1. Identification of Transposable Elements Represented on the Array

 The nonredundant sequences for the identified transposable elements (TEs) in wheat were downloaded from GrainGenes nrTREP database and used to query the Affymetrix wheat target sequences for homology with a cut-off of *e-*10. The two classes of transposable elements were well represented in the 476 unique TE sequences in the database that included 218 retrotransposons 182 DNA transposons, and 76 unclassified TEs.

The query identified 1,613 probesets on the wheat array representing 251 unique TEs with 75% retrotransposons and 23% DNA transposons (Table 2); we will refer to each probeset from now on to encode a TE. Among the retrotransposons, *Gypsy* and *Copia* were the most represented while the *CACTA* and *Mariner* were the most represented DNA transposons. Both *Gypsy* and *Copia* belong to the long-terminal repeat (LTR) containing family of retrotransposons wherein the autonomous element LTRs flank several genes needed for transposition. *CACTA* and Mariner are TEs flanked by short-terminal inverted repeats (TIR).

### 3.2. Expression of Wheat Transposable Elements in Response to Cold Treatment

Of the 1,613 TEs represented, 760 were expressed (Table 2), about 47% of all the TE probesets on the array. The majority of the expressed TEs were retrotransposons (529), 74% of which were of the *Gypsy* type. Of the 214 expressed DNA transposons 54% were *CACTA* elements.

**Figure 1:**
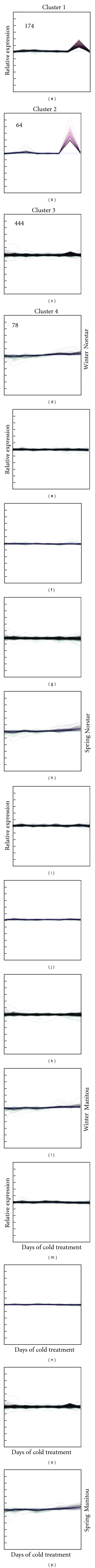
Pattern of TE gene expression during cold acclimation. The expression profiles of 760 TE genes across all 8 time points were grouped into 4 clusters using the *k*-means algorithm. Different numbers of groups were tested, but we found the grouping into 4 gave the best representation of the different profiles. The mean-centered relative gene expression value (in log_2_ scale) for each gene was plotted on the *y*-axis, and the time of cold treatment was plotted on the *x*-axis. Tick marks on the *x*-axis represent 0, 2, 14, 21, 28, 35, 42, 56, and 70 days of cold treatment. Tick marks for 0 and 70 days overlap with the sides of the cluster box. The value at the upper left corner on each of the 4 top panels indicates the number of TEs in the cluster.

Clustering of the profiles of the expressed TEs into 4 groups using the *k*-means algorithm showed that the majority of the TEs were expressed at a constant level throughout the duration of the experiment in three of the genotypes (Figure 1). In winter Norstar about 31% of the expressed TEs showed a burst of activity at 56 days of cold treatment, just after vernalization saturation was reached. This burst of TE expression, however, was rapidly repressed to basal level. TEs in cluster 1 showed a burst in relative expression between 2x and 4x above the basal level while Cluster 2 showed a burst of expression between 4x and 70x above the basal level. Expression of TEs in clusters 3 and 4 was similar to the other genotypes.

The TEs in clusters 1 and 2 (listed in Supplemental data 1 available online at doi:10.1155/2012/232530) included representatives from both Class I (45 members) and II (191 members). Of the Class I TEs, the superfamily *CACTA* had 14 families represented with family *Jorge* having the highest number of members (17). Among the Class II TEs, the superfamily *Gypsy* had 22 families represented. Four of the *Gypsy* families had 12 or more members (*Sabrina*-27, *Sumana*-21, *Wilma*-17, and *Fatima*-12). A member of the family *Sakura* had the highest change in expression (70x).

### 3.3. TE Expression and the *Vrn-A1* Locus

The spring Norstar genome is ~99.9% identical to winter Norstar, yet its TEs in cluster 1 and 2 did not exhibit the same burst in activity. The main difference between these two genotypes is the swapped *Vrn-A1* locus. Winter Norstar has the winter allele *vrn-A1* gene that requires cold treatment to be expressed, whereas spring Norstar has the spring allele *Vrn-A1* that is constitutively expressed and does not require vernalization to flower. This suggests that the *Vrn-A1* locus could be playing a role in the burst in TE activity in cluster 1 and 2 in winter Norstar.

 Winter Manitou and winter Norstar share the swapped region that includes the winter allele *vrn-A1*. Similar to winter Norstar, winter Manitou requires cold treatment to flower and reaches vernalization saturation after 42 days. However, the winter Manitou TEs in cluster 1 and 2 did not show the burst of activity observed in winter Norstar indicating that other factor(s) outside of the swapped region are required for the burst in TE expression.

### 3.4. Cluster 1 and 2 TE Expression Activated during Drought Stress

**Figure 2:**
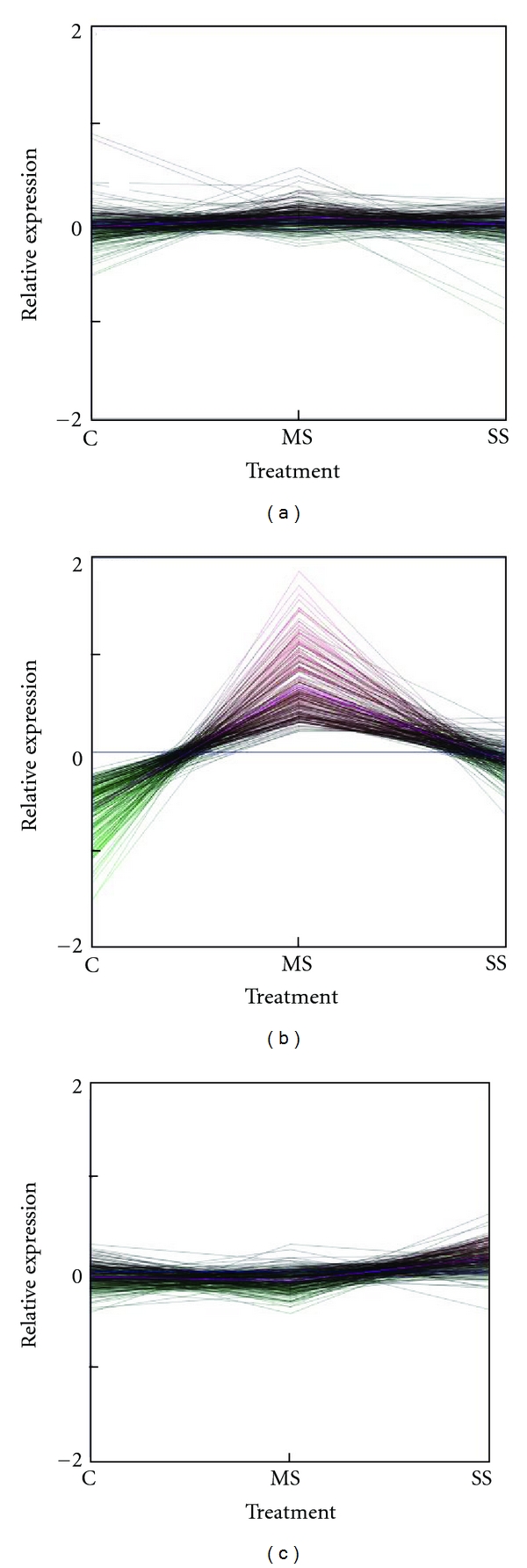
TE expression profiles during drought treatment. The expression profiles of 218 TE genes during drought stress in (a) bread wheat Chinese Spring, (b) Chinese Spring 5AL deletion line, and (c) durum wheat Cresco were compared. The mean-centered relative gene expression value (in log_2_ scale) for each gene was plotted on the *y*-axis and the level of drought treatment was indicated on the *x*-axis. Tick marks on the *x*-axis C, MS, and SS represent control, moderate stress, and severe stress, respectively.

In hexaploid wheat, a genomewide expression analysis during drought stress was previously carried out by Aprile et al. (2009 [12]) using the same Affymetrix genome chips used in our study. Their data showed high expression of a cluster of 91 genes only in the Chinese Spring 5AL-10 deletion line but not in the two other lines examined: wild type bread wheat Chinese Spring and durum wheat Cresco [12]. Of the 91 genes in the cluster, 13 were annotated as TE while the others were mainly of unknown function. Comparison of the probesets of the 13 transposable elements observed to be upregulated in the Chinese Spring 5AL-10 deletion line showed that these were a subset of the same transposable elements that were upregulated in winter Norstar in our study. We therefore examined the expression of the same 238 TEs that were upregulated in winter Norstar in the genotypes examined during drought stress using their dataset. The microarray data from the work of Aprile et al. was downloaded from the Plant Expression Database and reanalyzed to determine the expression profiles of the TEs in cluster 1 and 2 in our study. As shown in Figure 2, more than 90% of the TEs that were upregulated during cold treatment in our study were also upregulated in the Chinese Spring 5AL-10 deletion line during moderate drought treatment in a genotype-dependent manner. Chinese Spring 5AL-10 is derived from Chinese Spring wherein a segment of the long arm of chromosome 5 from 0.56 region to the end of the telomere was deleted. The deleted region contains several genes including those that affect low-temperature tolerance and flowering time, including the *Vrn-A1* locus. Thus, this data not only provides support to the genotype-dependent expression of TEs we observed in winter Norstar but further implicates the region containing the *Vrn-A1* locus in this process.

## 4. Discussion

### 4.1. Validation of the Burst of TE Expression in Winter Norstar

**Figure 3:**
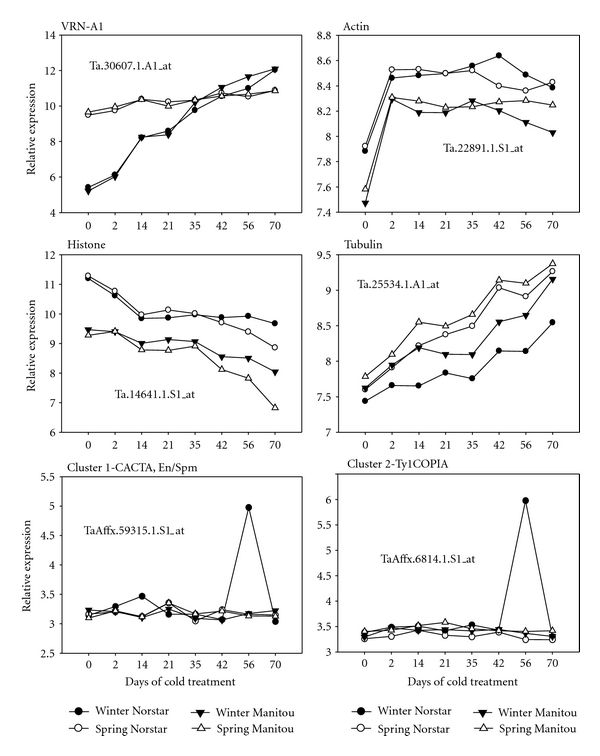
Expression profiles of selected TE and non-TE genes. The *y*-axis shows the values for the relative expression of the vernalization gene *VRN-A1*, selected housekeeping genes for actin, histone, and tubulin and representative TE from cluster 1 and 2 given as mean of log_2_-transformed signal intensities. The *x*-axis shows the days of cold treatment. The identification of each probeset is displayed on each chart.

Several lines of evidence support or validate the stage-specific burst of expression in winter Norstar. First, the signal and background levels in the biological samples at 56 days after treatment were similar to the other slides in the time-series experiment (see Supplemental data 2). The mean signal for each of the biological replicates at 56 days after cold treatment was between 6.13 and 6.14 (log_2_), similar to the rest of the samples in the whole experiment indicating that the increase in TE expression at this time point was not due to technical experimental errors. Furthermore, majority of the transposable elements in winter Norstar showed constant expression that was similar to the other genotypes. Only a subset of the transposons appears to show a burst of expression at 56 days after cold treatment. If the high signal observed in winter Norstar was an artifact, it is expected that most, if not all, of the TEs would show a higher signal at 56 days of cold treatment. Second, as we previously reported, analysis of the expression of known cold responsive genes using the same dataset gave expression profiles consistent with other independently published reports [15]. For example, the expression of the *Vrn-A1* gene in the swapped *Vrn-A1* locus showed the predicted swapped expression profiles in the spring and winter genotypes (Figure 3). As expected the spring *Vrn-A1* allele was constitutively expressed in the spring habit genotypes, whereas the *vrn-A1* winter allele showed a cold-inducible expression profile in the winter genotypes. Third, at 56 days after cold treatment in winter Norstar, the expression profiles of selected housekeeping genes commonly used as controls in other transcript level measurement methods were consistent with the trends in other genotypes (Figure 3). Lastly, genotype- and treatment-stage-specific induction of TEs have been previously reported in wheat (see above) and in other plants [14, 22, 23].

### 4.2. Genotype, and Developmental-Stage-Specific TE Expression in Plants

Recent reports have shown instances of genotype- and stage-specific expression of TEs in other plants. In alfalfa, a genotype-specific induction of a family of TEs during cold treatment was exhibited only in the most cold-hardy line investigated [14]. TEs in Arabidopsis pollen were also shown to be reactivated and transposed only in the vegetative nucleus but not in the sperm nuclei [22]. In rice, spaceflight-induced stress due to cosmic irradiation, microgravity, and space magnetic field [24] resulted in new *mPing* TE transpositions in two of the three cultivars grown in space [25]. The rice transposable element *mPing* has also been shown to be preferentially amplified in the genome of the rice strain EG4 compared to that of Nipponbare [26]. Furthermore, a survey of TE expression in rice during development revealed that the heading stage panicle had the highest detected expression, whereas the somatic shoot tissue had the lowest [23]. Thus, the observed genotype-dependent burst of TE expression after vernalization saturation in the crowns of cold-treated winter Norstar wheat in our report is not an isolated case.

### 4.3. Epigenetic Regulation of *Vrn1* and TEs

Flowering time is an epigenetically controlled process in higher plants [27–29]. In Arabidopsis, the model dicot plant system where the process has been investigated more intensively, epigenetic control includes both DNA methylation and chromatin remodeling. Recent advances indicate that vernalization, the process wherein plant exposure to non-freezing temperature accelerates the competence to flower, involves the Polycomb group (PcG) of genes (reviewed in [28]) in the silencing of FLOWERING LOCUS C (*FLC*). The expression of *FLC*, the critical gene in the vernalization response in Arabidopsis, is reduced as the level of methylation in lysine 27 of histone3 (H3K27me3) is progressively increased. In cereals, the *Vrn-A1* gene which controls flowering time is also epigenetically regulated [28, 30]. In barley, it has been shown that the repressed HvVRN1 has high level of H3K27me3 before vernalization. Vernalization-induced activation of the gene correlated with an increased level of methylation in lysine 4 of histone 3 (H3K4me3) and a decrease in H3K27me3 in the HvVRN1 chromatin. It has been postulated that the epigenetic memory of vernalization in cereals is based on the maintenance of an active chromatin state of the *Vrn1* locus [28].

Flowering time is also accelerated by environmental stress [29, 31]. Like TE activation, the epigenetic mechanisms of flowering time acceleration include DNA methylation, histone modifications, and microRNAs (reviewed in [30]). In this study, the burst in TE expression during cold treatment in the crowns of winter Norstar coincided with the stage after vernalization saturation has been achieved. Vernalization saturation is reached when the shoot apex has transitioned from vegetative to reproductive state (VTR); thus, additional cold treatment no longer accelerates flowering. Do the mechanisms that control flowering time during stress overlap with the activation of TEs? Is the observed burst of TE reactivation simply a response to the same epigenetic mechanisms that control flowering time?

Does the *Vrn-A1* locus play a role in the observed burst in TE expression? If so, then why was the same phenomenon not observed in winter Manitou which shares the same winter allele *vrn-A1* locus as winter Norstar? It is possible that both the region encoding the winter allele *vrn-A1* and genetic factors specific to Norstar background but outside the swapped *vrn-A1* locus are required for this burst of TE expression. Thus, the burst of TE expression was not observed even in spring Norstar which shares ~99.9% of winter Norstar genome but carries the spring *Vrn-A1* allele. The genotype-dependent reactivation of the same TEs during drought stress in Chinese Spring 5AL-10 line where the *Vrn-A1* was deleted supports the potential involvement of *Vrn-A1* locus in this phenomenon. Other factors that maybe involved in the epigenetic regulation of TE expression aside from the *Vrn-A1* locus may include those that encode for DNA-methylases, DNA-demethylases, small RNAs, and the proteins involved in small RNA processing, all of which have been implicated in chromatin remodeling [1]. Genetic variations in these loci between Norstar and winter Manitou background could be responsible for the differences in the expression of TEs during cold acclimation. Clearly, more tests are needed to determine whether these factors and the *Vrn1* locus are linked to the observed genotype-dependent TE burst of activation in wheat.

### 4.4. TE and Evolution

The stress-associated reactivation of TEs is postulated to play a role in host genome plasticity to survive adverse environments [32, 33]. Stress-induced TE mobility increases the generation of genetic variability that can be useful for adaption. In support of this, the rice *mPing* MITEs have been shown to be preferentially amplified in domesticated cultivars adapted to environmental extremes [34]. Furthermore, promoters of some transposable elements show similarity to those found in regulatory regions of host defense genes [5].

 The molecular bases of the mechanisms involved in TE repression and reactivation in plants are beginning to unfold but are still not clearly understood. Transposable elements make up a substantial fraction of plant genomes and have been shown to serve as a major contributor of genetic variations. How the host suppresses and reactivates the expression of TEs in its genome is a key issue in genome biology. It has been proposed that the theory of punctuated equilibria [35, 36] in evolutionary biology results from the epigenetic control of TE expression [33]. Our data provides insights into how TEs are regulated *in planta*. Our result indicates that TE expression in wheat could be induced in a genotype and developmental stage specific manner during cold treatment and suggests a potential role of the *Vrn-A1* locus in this process.

## Supplementary Material

The Supplemental data includes (1) List of 238 probesets (Affymetrix Wheat GenomeChip) representing transposable elements and (2) the Box-plots of raw signal and normalized intensities in the 96 microarray slides used in the experiment.

## Figures and Tables

**Table 1:** Vernalization genotype and minimum cold tolerance of near isogenic lines used in these studies.

Line	Genotype	LT50
Winter Norstar	vrn-A1, vrn-B1, vrn-D1	−23a
Spring Manitou	*Vrn-A1*, vrn-B1, vrn-D1	−8.3c
Winter Manitou	vrn-A1, vrn-B1, vrn-D1	−13.3b
Spring Norstar	*Vrn-A1*, vrn-B1, vrn-D1	−13b

LT50 indicates the lowest temperature by which 50% of the treated samples survived after acclimation; a to c: within columns, mean numbers followed by the same letter are not different as determined by Duncan's new multiple range test (*P* < 0.05).

**Table 2:** Transposable elements represented on the array.

DNA transposons	On array	Cluster 1	Cluster 2	Cluster 3	Cluster 4
Helitron	3	0	0	0	0
HAT	2	0	0	1	0
CACTA	236	33	9	59	14
Harbinger	12	2	0	6	1
Mutator-like	11	0	0	5	1
Mariner	111	1	0	55	24
Unknown	4	0	0	3	0

Subtotal	379 (23%)				

*Retrotransposons*					
Copia	243	31	9	56	4
Gypsy	896	102	46	216	29
LINE	27	1	0	0	0
Unknown	43	2	0	31	2

Subtotal	1209 (75%)				

Unclassified	25	2	0	12	3

Total	1613	174	64	444	78
