# Supplementary material for: Genotype-dependent Burst of Transposable Element Expression in Crowns of Hexaploid Wheat (Triticum aestivum L.) during Cold Acclimation
Source: Comp Funct Genomics. 2012 Feb 28;2012:232530. doi: 10.1155/2012/232530 (PMC3299314; doi:10.1155/2012/232530)
Supplement: Supplementary file 1 — The Supplemental data includes (1) List of 238 probesets (Affymetrix Wheat GenomeChip) representing transposable elements and (2) the Box-plots of raw signal and normalized intensities in the 96 microarray slides used in the experiment. [file 232530.f1.pdf]

Supplemental Data 1

No.	Input Probe Set	Cluster	Expression	TE annotation
1	Ta.14430.1.S1_x_at	1	2.39	Retrotransposon, LTR, Gypsy, "RLG_Cereba_AY040832-1"
2	Ta.14430.3.A1_at	1	3.10	Retrotransposon, LTR, Gypsy, "RLG_Cereba_AY040832-2"
3	Ta.14586.1.A1_at	1	2.11	Retrotransposon, LTR, Gypsy, "RLG_Sumaya_59e04-1"
4	Ta.14586.2.A1_at	1	1.84	Retrotransposon, LTR, Gypsy, "RLG_Sumaya_116F2-1"
5	Ta.15005.1.S1_x_at	1	2.84	DNA transposon, TIR, CACTA, "DTC_TAT5_464G14-1"
6	Ta.16853.1.S1_at	1	2.31	Retrotransposon, LTR, Gypsy, "RLG_Erika_consensus-1"
7	Ta.17090.1.S1_at	1	3.31	Retrotransposon, LTR, Gypsy, "RLG_Sabrina_D_AY146588-2"
8	Ta.18921.1.S1_at	1	3.53	Retrotransposon, LTR, unknown, "RLX_Xalax_9p13-1" solo-LTR
9	Ta.19680.1.A1_at	1	1.97	Retrotransposon, LTR, Gypsy, "RLG_Sakura_10k23-4"
10	Ta.20007.1.S1_at	1	1.77	Retrotransposon, LTR, Gypsy, "RLG_Sumaya_59e04-1"
11	Ta.24083.1.A1_at	1	3.14	Retrotransposon, LTR, Gypsy, "RLG_Sabrina_C_AY494981-4"
12	Ta.25001.1.A1_x_at	1	1.99	Retrotransposon, LTR, Gypsy, "RLG_Sumaya_116F2-1"
13	Ta.25005.1.A1_x_at	1	1.62	DNA transposon, TIR, CACTA, "DTC_Clifford_consensus-1"
14	Ta.30918.1.S1_at	1	2.03	Retrotransposon, LTR, Gypsy, "RLG_Egug_EF067844-16"
15	Ta.3312.2.S1_at	1	1.81	Retrotransposon, LTR, Gypsy, "RLG_Sumaya_59e04-1"
16	TaAffx.105253.1.S1_x_at	1	2.93	Retrotransposon, LTR, Gypsy, "RLG_Quinta_10k23-1"
17	TaAffx.105253.2.S1_x_at	1	2.73	Retrotransposon, LTR, Gypsy, "RLG_Quinta_10k23-1"
18	TaAffx.106461.1.S1_at	1	3.93	Retrotransposon, LTR, Gypsy, "RLG_Derami_AY368673-1"
19	TaAffx.108177.1.S1_at	1	2.60	Retrotransposon, LTR, Gypsy, "RLG_Romani_B_64a5-1"
20	TaAffx.108993.1.S1_at	1	2.00	Retrotransposon, LTR, Gypsy, "RLG_Derami_AY368673-1"
21	TaAffx.109030.1.S1_at	1	1.83	DNA transposon, TIR, CACTA, "DTC_Jorge_59e04-1"
22	TaAffx.109064.1.S1_at	1	2.37	unknown, unknown, unknown, "XXX_unnamed_Z75561-1"
23	TaAffx.109279.1.S1_at	1	2.91	Retrotransposon, LTR, Gypsy, "RLG_Fatima_B_consensus-1"
24	TaAffx.109695.1.S1_at	1	2.06	DNA transposon, TIR, CACTA, "DTC_Pavel_AY188332-1"
25	TaAffx.110026.1.S1_at	1	2.11	DNA transposon, TIR, CACTA, "DTC_Jude_AF446141-1"
26	TaAffx.110026.1.S1_x_at	1	1.68	DNA transposon, TIR, CACTA, "DTC_Jude_AF446141-1"
27	TaAffx.110794.1.S1_at	1	3.90	DNA transposon, TIR, CACTA, "DTC_Pavel_AY188332-1"
28	TaAffx.111080.1.S1_at	1	1.79	DNA transposon, TIR, CACTA, "DTC_Jorge_59e04-1"
29	TaAffx.111458.1.S1_at	1	1.89	Retrotransposon, LTR, Gypsy, "RLG_Sumana_59e04-2"
30	TaAffx.112335.1.S1_at	1	2.11	DNA transposon, TIR, CACTA, "DTC_Conan_consensus-1"
31	TaAffx.112844.1.S1_x_at	1	2.92	Retrotransposon, LTR, Copia, "RLC_WIS_A_consensus-1"
32	TaAffx.112844.3.S1_at	1	3.32	Retrotransposon, LTR, Copia, "RLC_WIS_A_consensus-1"
33	TaAffx.112844.3.S1_x_at	1	3.39	Retrotransposon, LTR, Copia, "RLC_WIS_A_consensus-1"
34	TaAffx.113761.1.S1_at	1	2.57	DNA transposon, TIR, CACTA, "DTC_Jorge_231A16-1"
35	TaAffx.113924.1.S1_at	1	1.97	Retrotransposon, LTR, Gypsy, "RLG_Laura_consensus-1"
36	TaAffx.113929.1.S1_at	1	2.03	DNA transposon, TIR, CACTA, "DTC_Pavel_AY188332-1"
37	TaAffx.120205.1.S1_at	1	3.73	DNA transposon, TIR, CACTA, "DTC_Balduin_consensus-1"
38	TaAffx.120212.2.S1_at	1	2.12	Retrotransposon, LTR, Gypsy, "RLG_Laura_609E6-1"
39	TaAffx.120239.1.S1_at	1	1.88	Retrotransposon, LTR, Copia, "RLC_WIS_A_consensus-1"
40	TaAffx.120239.1.S1_x_at	1	1.96	Retrotransposon, LTR, Copia, "RLC_WIS_A_consensus-1"
41	TaAffx.120260.1.S1_at	1	2.45	Retrotransposon, LTR, Gypsy, "RLG_WHAM_consensus-1"
42	TaAffx.124275.72.S1_at	1	1.78	Retrotransposon, LTR, Gypsy, "RLG_Wilma_AY146588-3"
43	TaAffx.128541.37.S1_x_at	1	2.13	Retrotransposon, LTR, Gypsy, "RLG_Sabrina_C_AY494981-4"
44	TaAffx.128541.53.S1_at	1	1.87	Retrotransposon, LTR, Gypsy, "RLG_Egug_AY268139-1"
45	TaAffx.128541.55.S1_at	1	2.22	Retrotransposon, LTR, Gypsy, "RLG_Sabrina_B_AY368673-1"
46	TaAffx.128541.6.S1_at	1	3.42	Retrotransposon, LTR, Gypsy, "RLG_Sabrina_D_AY146588-2"
47	TaAffx.128541.9.S1_x_at	1	2.43	Retrotransposon, LTR, Gypsy, "RLG_Sabrina_B_AY368673-1"
48	TaAffx.134909.1.S1_at	1	1.80	Retrotransposon, LTR, Gypsy, "RLG_Sumana_59e04-2"
49	TaAffx.137.1.S1_at	1	1.78	DNA transposon, TIR, CACTA, "DTC_Clifford_consensus-1"
50	TaAffx.16070.1.S1_at	1	1.78	DNA transposon, TIR, CACTA, "DTC_Jorge_59e04-1"
51	TaAffx.1861.5.S1_x_at	1	2.69	Retrotransposon, LTR, Copia, "RLC_WIS_A_consensus-1"
52	TaAffx.1861.9.S1_x_at	1	2.16	Retrotransposon, LTR, Copia, "RLC_WIS_B_consensus-1"
53	TaAffx.22.1.A1_at	1	1.90	DNA transposon, TIR, Mariner, "DTT_Thalos_consensus-1"
54	TaAffx.23084.3.S1_at	1	2.38	Retrotransposon, LTR, Gypsy, "RLG_Quinta_10k23-1"
55	TaAffx.23084.3.S1_x_at	1	3.76	Retrotransposon, LTR, Gypsy, "RLG_Quinta_10k23-1"
56	TaAffx.23114.1.S1_at	1	2.07	Retrotransposon, LTR, Gypsy, "RLG_Fatima_B_consensus-1"
57	TaAffx.23129.1.S1_at	1	2.01	DNA transposon, TIR, CACTA, "DTC_Jorge_231A16-1"
58	TaAffx.23589.1.S1_at	1	2.00	DNA transposon, TIR, CACTA, "DTC_Jorge_59e04-1"
59	TaAffx.24523.1.S1_at	1	1.72	Retrotransposon, LTR, Gypsy, "RLG_Quinta_10k23-1"
60	TaAffx.24531.1.S1_at	1	2.58	Retrotransposon, LTR, Gypsy, "RLG_Fatima_consensus-1"
61	TaAffx.25548.1.S1_at	1	1.94	Retrotransposon, LTR, Gypsy, "RLG_Sumana_59e04-2"

62	TaAffx.25575.1.S1_at	1	2.01	Retrotransposon, LTR, Gypsy, "RLG_WHAM_consensus-1"
63	TaAffx.25679.1.S1_at	1	2.23	Retrotransposon, LTR, Copia, "RLC_Angela_B_consensus-1"
64	TaAffx.25679.1.S1_x_at	1	2.18	Retrotransposon, LTR, Copia, "RLC_Angela_B_consensus-1"
65	TaAffx.25683.1.S1_at	1	1.67	Retrotransposon, LTR, Gypsy, "RLG_Sakura_115G1-1"
66	TaAffx.25699.1.S1_at	1	1.84	DNA transposon, TIR, CACTA, "DTC_Jorge_59e04-1"
67	TaAffx.25951.1.S1_at	1	2.95	Retrotransposon, LTR, Copia, "RLC_WIS_A_consensus-1"
68	TaAffx.26038.1.S1_at	1	1.68	Retrotransposon, LTR, Copia, "RLC_Angela_A_consensus-1"
69	TaAffx.26629.1.S1_at	1	2.22	Retrotransposon, LTR, Gypsy, "RLG_Romana_AF459088-2"
70	TaAffx.27006.1.S1_at	1	3.85	Retrotransposon, LTR, Copia, "RLC_Angela_B_consensus-1"
71	TaAffx.27144.1.S1_at	1	3.57	DNA transposon, TIR, Harbinger, "DTH_Orpheus_consensus-1"
72	TaAffx.27399.1.S1_at	1	1.70	Retrotransposon, LTR, Copia, "RLC_TAR2_AY853252-1" ?
73	TaAffx.28165.1.S1_at	1	2.11	Retrotransposon, LTR, Gypsy, "RLG_Sakura_115G1-1"
74	TaAffx.29078.1.S1_at	1	2.46	DNA transposon, TIR, Harbinger, "DTH_Kerberos_consensus-1"
75	TaAffx.29302.1.S1_at	1	2.04	Retrotransposon, LTR, Copia, "RLC_WIS_B_consensus-1"
76	TaAffx.29362.1.S1_at	1	2.65	Retrotransposon, LTR, Copia, "RLC_Angela_A_consensus-1"
77	TaAffx.29664.1.S1_at	1	1.76	Retrotransposon, LTR, Gypsy, "RLG_Fatima_B_consensus-1" ,
78	TaAffx.30466.1.S1_at	1	1.63	Retrotransposon, LTR, Gypsy, "RLG_Wilma_AY494981-2"
79	TaAffx.30561.1.S1_at	1	2.16	Retrotransposon, LTR, Gypsy, "RLG_Laura_609E6-1"
80	TaAffx.30561.1.S1_x_at	1	1.73	Retrotransposon, LTR, Gypsy, "RLG_Laura_609E6-1"
81	TaAffx.30783.1.S1_at	1	2.48	Retrotransposon, LTR, Gypsy, "RLG_BAGY2_consensus-1"
82	TaAffx.31309.1.S1_at	1	2.86	DNA transposon, TIR, CACTA, "DTC_Jorge_59e04-1"
83	TaAffx.31410.1.S1_at	1	2.07	Retrotransposon, LTR, Gypsy, "RLG_Sumaya_59e04-1"
84	TaAffx.31443.1.S1_at	1	2.00	Retrotransposon, LTR, Gypsy, "RLG_Sumana_59e04-2"
85	TaAffx.31501.1.S1_at	1	3.41	Retrotransposon, LTR, Gypsy, "RLG_Sakura_115G1-1"
86	TaAffx.31539.1.S1_at	1	1.81	Retrotransposon, LTR, Copia, "RLC_Sasanda_EU157184-1"
87	TaAffx.31561.1.S1_at	1	2.51	Retrotransposon, LTR, Gypsy, "RLG_Romani_AF459088-1"
88	TaAffx.31839.1.S1_at	1	1.65	Retrotransposon, LTR, Gypsy, "RLG_Wilma_AY494981-2"
89	TaAffx.31867.1.S1_at	1	2.64	Retrotransposon, LTR, Gypsy, "RLG_Egug_EF067844-16"
90	TaAffx.31972.1.S1_at	1	2.51	Retrotransposon, LTR, Gypsy, "RLG_Danae_consensus-1"
91	TaAffx.47708.1.S1_at	1	1.76	DNA transposon, TIR, CACTA, "DTC_Jorge_AF326781-1"
92	TaAffx.53280.1.S1_at	1	2.68	Retrotransposon, LTR, Copia, "RLC_Angela_A_consensus-1"
93	TaAffx.53317.1.S1_at	1	2.21	Retrotransposon, LTR, Gypsy, "RLG_Sabrina_consensus-1"
94	TaAffx.54404.1.S1_at	1	2.62	Retrotransposon, LTR, Copia, "RLC_Valerie_AY661558-2"
95	TaAffx.54410.1.S1_at	1	1.64	Retrotransposon, LTR, Gypsy, "RLG_Carmilla_consensus-1"
96	TaAffx.54439.1.S1_at	1	2.04	Retrotransposon, LTR, Gypsy, "RLG_Latidu_10k23-1"
97	TaAffx.54442.1.S1_at	1	2.06	DNA transposon, TIR, CACTA, "DTC_Sherlock_consensus-1"
98	TaAffx.54490.1.S1_at	1	2.79	Retrotransposon, LTR, Gypsy, "RLG_Sabrina_C_210J24-2"
99	TaAffx.54534.1.S1_at	1	1.96	DNA transposon, TIR, CACTA, "DTC_Jorge_59e04-1"
100	TaAffx.56188.1.S1_at	1	2.00	Retrotransposon, LTR, Gypsy, "RLG_Wilma_AY494981-2"
101	TaAffx.56188.1.S1_x_at	1	2.09	Retrotransposon, LTR, Gypsy, "RLG_Wilma_AY494981-2"
102	TaAffx.56371.1.S1_at	1	2.38	DNA transposon, TIR, CACTA, "DTC_TAT5_464G14-1"
103	TaAffx.56411.1.S1_at	1	3.67	Retrotransposon, LTR, Gypsy, "RLG_Sabrina_B_AY368673-1"
104	TaAffx.56527.1.S1_at	1	2.78	Retrotransposon, LTR, Copia, "RLC_WIS_B_consensus-1"
105	TaAffx.56527.1.S1_x_at	1	3.46	Retrotransposon, LTR, Copia, "RLC_WIS_B_consensus-1"
106	TaAffx.56833.1.S1_at	1	2.15	Retrotransposon, LTR, Gypsy, "RLG_Sabrina_B_AY368673-1"
107	TaAffx.56902.1.S1_at	1	1.88	DNA transposon, TIR, CACTA, "DTC_Jorge_AF326781-1"
108	TaAffx.57036.1.S1_at	1	2.34	Retrotransposon, LTR, Gypsy, "RLG_Lila_42j2-3"
109	TaAffx.58148.1.S1_at	1	2.13	Retrotransposon, LTR, Gypsy, "RLG_Romani_10k23-3"
110	TaAffx.58384.1.S1_at	1	1.77	Retrotransposon, LTR, Copia, "RLC_WIS_A_consensus-1"
111	TaAffx.58444.1.S1_at	1	3.15	Retrotransposon, LTR, Gypsy, "RLG_Cereba_AY040832-1"
112	TaAffx.58457.1.S1_x_at	1	2.85	Retrotransposon, LTR, Gypsy, "RLG_Sabrina_D_AY146588-2"
113	TaAffx.58636.1.S1_at	1	2.05	Retrotransposon, LTR, Gypsy, "RLG_Derami_A_AY188333-1"
114	TaAffx.58770.1.S1_at	1	2.01	Retrotransposon, LTR, Gypsy, "RLG_Fatima_consensus-1"
115	TaAffx.58779.1.S1_at	1	3.57	Retrotransposon, LTR, Gypsy, "RLG_Wilma_AY494981-2"
116	TaAffx.59315.1.S1_at	1	3.83	DNA transposon, TIR, CACTA, "DTC_Conan_consensus-1"
117	TaAffx.59390.1.S1_at	1	2.51	DNA transposon, TIR, CACTA, "DTC_Jorge_59e04-1"
118	TaAffx.5942.1.S1_at	1	2.52	DNA transposon, TIR, CACTA, "DTC_Balduin_consensus-1"
119	TaAffx.64697.1.S1_at	1	2.48	Retrotransposon, LTR, Gypsy, "RLG_Laura_consensus-1"
120	TaAffx.65606.1.S1_at	1	3.14	Retrotransposon, LTR, Copia, "RLC_WIS_B_consensus-1"
121	TaAffx.65702.1.S1_at	1	1.97	Retrotransposon, LTR, Gypsy, "RLG_Sumaya_59e04-1"
122	TaAffx.6588.1.S1_at	1	2.37	Retrotransposon, LTR, Copia, "RLC_Maximus_consensus-1"
123	TaAffx.6630.1.S1_at	1	1.71	Retrotransposon, LTR, Gypsy, "RLG_Fatima_B_consensus-1"
124	TaAffx.6657.1.S1_at	1	1.68	Retrotransposon, LTR, Gypsy, "RLG_Fatima_B_consensus-1"
125	TaAffx.6759.1.S1_at	1	1.69	DNA transposon, TIR, CACTA, "DTC_Conan_consensus-1"

126	TaAffx.69933.5.A1_at	1	2.81	Retrotransposon, LTR, Copia, "RLC_WIS_A_consensus-1"
127	TaAffx.70061.12.S1_at	1	2.24	Retrotransposon, LTR, Gypsy, "RLG_Erika_consensus-1"
128	TaAffx.70061.14.A1_at	1	2.33	Retrotransposon, LTR, Gypsy, "RLG_Sumaya_116F2-1"
129	TaAffx.70061.17.S1_at	1	1.88	Retrotransposon, LTR, Gypsy, "RLG_Sumaya_59e04-1"
130	TaAffx.70061.28.S1_at	1	4.04	Retrotransposon, LTR, Gypsy, "RLG_Sumaya_59e04-1"
131	TaAffx.70061.8.S1_at	1	2.03	Retrotransposon, LTR, Gypsy, "RLG_Sumana_59e04-2"
132	TaAffx.7031.1.S1_at	1	2.25	Retrotransposon, LTR, Gypsy, "RLG_Wilma_AY146588-3"
133	TaAffx.70805.1.A1_at	1	1.65	DNA transposon, TIR, CACTA, "DTC_Enac_AY146588-1"
134	TaAffx.7157.1.S1_at	1	2.51	Retrotransposon, LTR, Gypsy, "RLG_Sabrina_C_210J24-2"
135	TaAffx.74416.6.A1_at	1	3.18	Retrotransposon, LTR, Gypsy, "RLG_Sabrina_C_210J24-2"
136	TaAffx.76.4.S1_at	1	2.53	Retrotransposon, LTR, Gypsy, "RLG_Egug_AY268139-1"
137	TaAffx.76.4.S1_x_at	1	4.10	Retrotransposon, LTR, Gypsy, "RLG_Egug_AY268139-1"
138	TaAffx.7624.1.S1_at	1	2.02	Retrotransposon, LTR, Gypsy, "RLG_Derami_AY368673-1"
139	TaAffx.7664.1.S1_at	1	2.11	Retrotransposon, LTR, unknown, "RLX_Xalax_9p13-1" solo-LTR
140	TaAffx.7704.1.S1_at	1	3.88	DNA transposon, TIR, CACTA, "DTC_Pavel_AY188332-1"
141	TaAffx.77470.1.A1_at	1	3.88	unknown, unknown, unknown, "XXX_Iapetus_103H9-1"
142	TaAffx.78045.1.S1_at	1	3.95	DNA transposon, TIR, CACTA, "DTC_Byron_AY146587-2"
143	TaAffx.78249.1.S1_at	1	2.21	Retrotransposon, LINE, unknown, "RIX_Stasy_294D11-1"
144	TaAffx.7866.1.S1_at	1	1.87	Retrotransposon, LTR, Gypsy, "RLG_Laura_609E6-1"
145	TaAffx.79902.1.S1_at	1	2.25	DNA transposon, TIR, CACTA, "DTC_TAT1_AF459088-1"
146	TaAffx.80312.1.S1_x_at	1	1.95	Retrotransposon, LTR, Gypsy, "RLG_Sabrina_C_AY494981-4"
147	TaAffx.80328.1.S1_at	1	1.81	DNA transposon, TIR, CACTA, "DTC_Boris_59e04-1"
148	TaAffx.80366.1.S1_at	1	1.84	Retrotransposon, LTR, Copia, "RLC_Angela_B_consensus-1"
149	TaAffx.80413.1.S1_at	1	2.15	Retrotransposon, LTR, Gypsy, "RLG_Fatima_consensus-1"
150	TaAffx.8047.1.S1_at	1	3.50	Retrotransposon, LTR, Gypsy, "RLG_Egug_AY268139-1"
151	TaAffx.80578.1.S1_at	1	3.39	Retrotransposon, LTR, Gypsy, "RLG_Wilma_AY494981-2"
152	TaAffx.80928.1.S1_at	1	2.50	Retrotransposon, LTR, Gypsy, "RLG_Laura_609E6-1"
153	TaAffx.82073.1.S1_at	1	3.07	Retrotransposon, LTR, Copia, "RLC_Maximus_consensus-1"
154	TaAffx.82125.1.S1_at	1	1.80	Retrotransposon, LTR, Gypsy, "RLG_Sabrina_D_115G1-2"
155	TaAffx.82624.1.S1_at	1	2.97	Retrotransposon, LTR, Gypsy, "RLG_Sabrina_D_AY146588-2"
156	TaAffx.83311.1.S1_x_at	1	2.82	Retrotransposon, LTR, Gypsy, "RLG_Sumana_59e04-2"
157	TaAffx.83778.1.S1_at	1	3.80	Retrotransposon, LTR, Copia, "RLC_WIS_A_consensus-1"
158	TaAffx.83873.1.S1_at	1	1.84	Retrotransposon, LTR, Gypsy, "RLG_Sabrina_C_AY494981-4"
159	TaAffx.83915.1.S1_at	1	2.41	Retrotransposon, LTR, Gypsy, "RLG_Romana_AF459088-2"
160	TaAffx.83941.1.S1_at	1	3.18	DNA transposon, TIR, CACTA, "DTC_Clifford_consensus-1"
161	TaAffx.84030.1.S1_at	1	1.80	Retrotransposon, LTR, Gypsy, "RLG_Olivia_10k23-1"
162	TaAffx.84031.1.S1_at	1	1.68	Retrotransposon, LTR, Gypsy, "RLG_Fatima_consensus-1"
163	TaAffx.84049.1.S1_at	1	2.31	Retrotransposon, LTR, Gypsy, "RLG_Laura_609E6-1"
164	TaAffx.84323.1.S1_at	1	2.51	Retrotransposon, LTR, Gypsy, "RLG_Romana_AF459088-2"
165	TaAffx.85099.1.S1_at	1	2.12	Retrotransposon, LTR, Gypsy, "RLG_Nusif_AY494981-1"
166	TaAffx.86127.1.S1_at	1	1.64	Retrotransposon, LTR, Copia, "RLC_Maximus_consensus-1"
167	TaAffx.8798.1.S1_at	1	1.86	Retrotransposon, LTR, Gypsy, "RLG_Fatima_consensus-1"
168	TaAffx.89491.1.S1_at	1	1.92	Retrotransposon, LTR, Copia, "RLC_Maximus_consensus-1"
169	TaAffx.8981.1.S1_at	1	2.43	Retrotransposon, LTR, Gypsy, "RLG_Wilma_AY494981-2"
170	TaAffx.9030.1.S1_at	1	2.96	Retrotransposon, LTR, Copia, "RLC_Angela_B_consensus-1"
171	TaAffx.93242.1.A1_at	1	1.97	Retrotransposon, LTR, Copia, "RLC_unnamed_42j2-1" fragment
172	TaAffx.97265.1.S1_x_at	1	2.78	Retrotransposon, LTR, Gypsy, "RLG_Fatima_B_consensus-1"
173	TaAffx.97289.1.S1_at	1	3.31	Retrotransposon, LTR, Gypsy, "RLG_Laura_consensus-1"
174	TaAffx.9856.1.S1_at	1	3.55	Retrotransposon, LTR, Gypsy, "RLG_Sabrina_D_AY146588-2"
175	Ta.20297.1.S1_at	2	25.09	Retrotransposon, LTR, Gypsy, "RLG_WHAM_consensus-1"
176	Ta.21696.1.S1_at	2	9.47	Retrotransposon, LTR, Gypsy, "RLG_Romana_AY494981-1"
177	TaAffx.105253.2.S1_at	2	4.23	Retrotransposon, LTR, Gypsy, "RLG_Quinta_10k23-1"
178	TaAffx.108987.1.S1_at	2	12.21	Retrotransposon, LTR, Gypsy, "RLG_Wilma_AY494981-2"
179	TaAffx.109002.1.S1_at	2	5.01	DNA transposon, TIR, CACTA, "DTC_Jorge_59e04-1"
180	TaAffx.109079.1.S1_at	2	6.73	DNA transposon, TIR, CACTA, "DTC_Clifford_consensus-1"
181	TaAffx.109268.1.S1_at	2	13.45	Retrotransposon, LTR, Gypsy, "RLG_Wilma_AY494981-2"
182	TaAffx.109268.1.S1_x_at	2	6.41	Retrotransposon, LTR, Gypsy, "RLG_Wilma_AY146588-3"
183	TaAffx.110754.1.S1_at	2	13.11	Retrotransposon, LTR, Gypsy, "RLG_Sumana_59e04-2"
184	TaAffx.111008.1.S1_at	2	5.45	Retrotransposon, LTR, Gypsy, "RLG_Sabrina_B_AY368673-1"
185	TaAffx.112770.1.S1_at	2	5.27	Retrotransposon, LTR, Copia, "RLC_Angela_B_consensus-1"
186	TaAffx.112770.1.S1_x_at	2	9.36	Retrotransposon, LTR, Copia, "RLC_Angela_B_consensus-1"
187	TaAffx.113515.1.S1_at	2	8.44	DNA transposon, TIR, CACTA, "DTC_Jorge_AF326781-1"
188	TaAffx.128541.51.S1_at	2	7.79	Retrotransposon, LTR, Gypsy, "RLG_Sabrina_B_AY368673-1"
189	TaAffx.128541.59.A1_at	2	7.45	Retrotransposon, LTR, Gypsy, "RLG_Sabrina_C_AY494981-4"

190	TaAffx.128541.69.S1_at	2	20.36	Retrotransposon, LTR, Gypsy, "RLG_Sabrina_D_AY146588-2"
191	TaAffx.128541.69.S1_x_at	2	18.90	Retrotransposon, LTR, Gypsy, "RLG_Sabrina_D_AY146588-2"
192	TaAffx.133843.2.S1_at	2	6.73	Retrotransposon, LTR, Gypsy, "RLG_Sabrina_D_115G1-2"
193	TaAffx.23084.1.S1_at	2	8.71	Retrotransposon, LTR, Gypsy, "RLG_Quinta_10k23-1"
194	TaAffx.23084.1.S1_x_at	2	9.28	Retrotransposon, LTR, Gypsy, "RLG_Quinta_10k23-1"
195	TaAffx.23114.2.S1_at	2	5.30	Retrotransposon, LTR, Gypsy, "RLG_Fatima_B_consensus-1" ,
196	TaAffx.23214.1.S1_at	2	11.82	Retrotransposon, LTR, Gypsy, "RLG_Cereba_AY040832-2"
197	TaAffx.24514.2.S1_at	2	8.15	Retrotransposon, LTR, Gypsy, "RLG_Cereba_AY040832-2"
198	TaAffx.25602.1.S1_s_at	2	16.54	Retrotransposon, LTR, Copia, "RLC_Eugene_consensus-1"
199	TaAffx.27004.1.S1_at	2	4.65	DNA transposon, TIR, CACTA, "DTC_Fergat_42j2-4"
200	TaAffx.27015.1.S1_at	2	4.39	Retrotransposon, LTR, Gypsy, "RLG_Cereba_AY040832-1"
201	TaAffx.27375.1.S1_at	2	12.67	DNA transposon, TIR, CACTA, "DTC_Jorge_AF326781-1"
202	TaAffx.28526.1.S1_at	2	5.08	Retrotransposon, LTR, Gypsy, "RLG_Romana_AY494981-1"
203	TaAffx.30272.3.A1_at	2	5.20	Retrotransposon, LTR, Copia, "RLC_Angela_B_consensus-1"
204	TaAffx.31295.1.S1_at	2	4.95	Retrotransposon, LTR, Gypsy, "RLG_Sabrina_consensus-1"
205	TaAffx.32091.1.S1_at	2	21.01	DNA transposon, TIR, CACTA, "DTC_Jorge_AF326781-1"
206	TaAffx.32098.1.S1_at	2	4.76	Retrotransposon, LTR, Gypsy, "RLG_Wilma_AY494981-2"
207	TaAffx.52994.1.S1_at	2	7.22	Retrotransposon, LTR, Copia, "RLC_Angela_B_consensus-1"
208	TaAffx.53736.1.S1_at	2	8.78	Retrotransposon, LTR, Gypsy, "RLG_Romani_10k23-3"
209	TaAffx.54173.1.S1_at	2	4.71	Retrotransposon, LTR, Gypsy, "RLG_Sakura_115G1-1"
210	TaAffx.54175.1.S1_at	2	4.95	Retrotransposon, LTR, Gypsy, "RLG_Sakura_10k23-4"
211	TaAffx.56355.1.S1_at	2	8.33	Retrotransposon, LTR, Gypsy, "RLG_Wilma_AY494981-2"
212	TaAffx.58547.1.S1_at	2	7.11	Retrotransposon, LTR, Copia, "RLC_Angela_B_consensus-1"
213	TaAffx.5865.2.A1_at	2	16.57	Retrotransposon, LTR, Gypsy, "RLG_Sakura_10k23-4"
214	TaAffx.59361.1.S1_at	2	4.52	Retrotransposon, LTR, Gypsy, "RLG_Sakura_10k23-4"
215	TaAffx.62172.1.S1_at	2	6.77	Retrotransposon, LTR, Copia, "RLC_WIS_B_consensus-1"
216	TaAffx.65642.1.S1_at	2	7.93	Retrotransposon, LTR, Gypsy, "RLG_WHAM_consensus-1"
217	TaAffx.65643.1.S1_at	2	4.90	DNA transposon, TIR, CACTA, "DTC_Jorge_59e04-1"
218	TaAffx.65647.1.S1_at	2	4.50	Retrotransposon, LTR, Copia, "RLC_WIS_A_consensus-1"
219	TaAffx.6672.1.S1_at	2	5.05	Retrotransposon, LTR, Gypsy, "RLG_Derami_A_AY188333-1"
220	TaAffx.6814.1.S1_at	2	6.40	Retrotransposon, LTR, Copia, "RLC_WIS_B_consensus-1"
221	TaAffx.70061.10.S1_at	2	4.51	Retrotransposon, LTR, Gypsy, "RLG_Sumana_59e04-2"
222	TaAffx.70061.16.S1_at	2	4.96	Retrotransposon, LTR, Gypsy, "RLG_Sumaya_59e04-1"
223	TaAffx.70061.29.S1_at	2	12.34	Retrotransposon, LTR, Gypsy, "RLG_Sumana_59e04-2"
224	TaAffx.70061.29.S1_x_at	2	10.89	Retrotransposon, LTR, Gypsy, "RLG_Sumana_59e04-2"
225	TaAffx.74416.2.S1_at	2	69.87	Retrotransposon, LTR, Gypsy, "RLG_Sakura_10k23-4"
226	TaAffx.80350.1.S1_at	2	10.29	Retrotransposon, LTR, Gypsy, "RLG_Wilma_AY146588-3"
227	TaAffx.80606.1.S1_at	2	12.57	Retrotransposon, LTR, Gypsy, "RLG_Wilma_AY146588-3"
228	TaAffx.80614.1.S1_at	2	6.43	Retrotransposon, LTR, Gypsy, "RLG_Egug_EF067844-16"
229	TaAffx.8194.1.S1_at	2	6.19	DNA transposon, TIR, CACTA, "DTC_Caspar_consensus-1"
230	TaAffx.83311.1.S1_at	2	5.24	Retrotransposon, LTR, Gypsy, "RLG_Sumana_59e04-2"
231	TaAffx.84020.1.S1_at	2	7.72	Retrotransposon, LTR, Gypsy, "RLG_Romani_10k23-3"
232	TaAffx.84028.1.S1_at	2	12.46	Retrotransposon, LTR, Gypsy, "RLG_Sakura_10k23-4"
233	TaAffx.84236.2.S1_at	2	5.23	Retrotransposon, LTR, Gypsy, "RLG_Sabrina_D_AY146588-2"
234	TaAffx.84236.2.S1_x_at	2	4.92	Retrotransposon, LTR, Gypsy, "RLG_Sabrina_D_AY146588-2"
235	TaAffx.84244.1.S1_at	2	4.34	DNA transposon, TIR, CACTA, "DTC_TAT1_AF459088-1"
236	TaAffx.84870.1.S1_at	2	4.90	Retrotransposon, LTR, Gypsy, "RLG_Laura_609E6-1"
237	TaAffx.85688.1.S1_at	2	7.08	Retrotransposon, LTR, Gypsy, "RLG_Wilma_AY146588-3"
238	TaAffx.86667.1.S1_at	2	4.82	Retrotransposon, LTR, Gypsy, "RLG_Sumaya_116F2-1"
