# Supplementary material for: Genotype-dependent Burst of Transposable Element Expression in Crowns of Hexaploid Wheat (Triticum aestivum L.) during Cold Acclimation
Source: Comp Funct Genomics. 2012 Feb 28;2012:232530. doi: 10.1155/2012/232530 (PMC3299314; doi:10.1155/2012/232530)
Supplement: Supplementary file 2 [file 232530.f2.pdf]

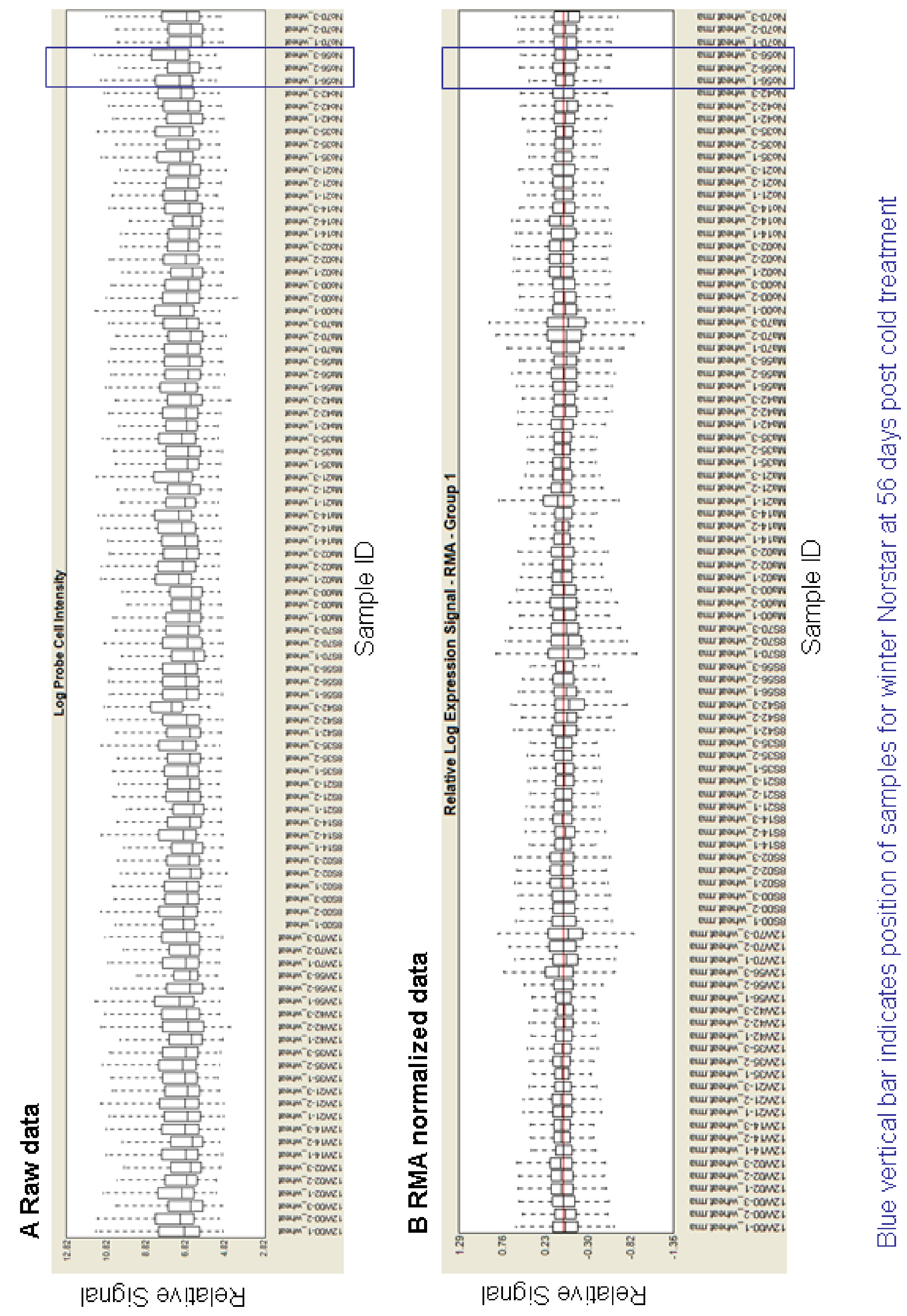

Supplemental Data 2

The box-plots of raw signal intensities (panel A) and normalized data (panel B) for each microarray slide show that the signals from the arrays for samples at 56 days after cold treatment are comparable to the rest of the slides.
